# TRPM7 is involved in acid‐induced necrotic cell death in a manner sensitive to progesterone in human cervical cancer cells

**DOI:** 10.14814/phy2.14157

**Published:** 2019-07-10

**Authors:** Tomohiro Numata, Kaori Sato‐Numata, Yasunobu Okada

**Affiliations:** ^1^ Department of Physiology, Graduate School of Medical Sciences Fukuoka University Fukuoka Japan; ^2^ Japan Society for the Promotion of Science Tokyo Japan; ^3^ Department of Molecular Cell Physiology Kyoto Prefectural University of Medicine Kyoto Japan; ^4^ Department of Cell Physiology National Institute for Physiological Sciences Okazaki Japan

**Keywords:** Acidotoxicity, apoptosis, cervical cancer cell, necrosis, progesterone, TRPM7

## Abstract

Because intravaginal pH is strongly acidic, it is important to investigate the effects of acidosis on cervical cancer cells. Recently, in response to strong acidosis, human cervical cancer HeLa cells were shown to exhibit necrosis after showing persistent cell swelling induced by Cl^−^ influx. Since cation influx should be accompanied with Cl^−^ influx to drive water inflow causing cell swelling, we here studied on the nature of acidotoxic cation conductance. The mRNA/protein expression was assessed by RT‐PCR and Western blotting. Ionic currents were measured by patch‐clamping techniques. Cell counting/viability and colorimetric assays were applied to assess proliferation rate and caspase 3/7 activity, respectively. Cell volume and size were measured by electronic sizing and video‐microscopic measurements, respectively. Acid exposure enhanced TRPM7 activity endogenously expressed in HeLa cells and exogenously overexpressed in HEK293T cells. Gene silencing of TRPM7 abolished acid‐induced cell swelling and necrosis but rather induced activation of apoptotic caspase 3/7 in HeLa cells. Overexpression with the pore charge‐neutralizing D1054A mutant suppressed acid‐enhanced cation currents, acid‐induced cell swelling, and acidotoxic necrosis in HEK293T cells. Progesterone treatment was surprisingly found to suppress molecular and functional expression of TRPM7 and cell proliferation in HeLa cells. Furthermore, in the progesterone‐treated cells, acid exposure did not induce persistent cell swelling followed by necrosis but induced persistent cell shrinkage and apoptotic cell death. These results indicate that in the human cervical cancer cells, TRPM7 is essentially involved in acidotoxic necrotic cell death, and progesterone inhibits TRPM7 expression thereby inhibiting acidotoxic necrosis by switching to apoptosis.

## Introduction

Acidosis is frequently observed in a number of pathological states including ischemic, inflammatory, hyperglycemic, and injurious situations (for Reviews: (Rehncrona, 1985; Chesler and Kaila, 1992; Siesjo, 1993; Lardner, 2001; Wemmie et al., 2006)) as well as the tumor microenvironments (for Reviews: (Vaupel et al., 1989; Lardner, 2001)). Even under physiological conditions, normal pH is between 4.0 and 5.0 in the human skin surface (Fluhr et al., 2001; Behne et al., 2002) and in the vagina of premenopausal women (Lang, 1955; Garcia‐Closas et al., 1999; Eschenbach et al., 2000). Strong acidosis may toxically contribute to cell death, called acidotoxicity, not only in brain neuronal and glial cells (Goldman et al., 1989; Siesjo et al., 1996; Ding et al., 2000) but also in epithelial cells (Argenzio and Eisemann, 1996). Acidotoxic cell death is necrotic in nature and is usually preceded by persistent cell swelling, called the necrotic volume increase (NVI), which is induced by water inflow driven by Na^+^ and Cl^−^ influx (for Reviews: (Okada et al., 2001, 2019)). The acidotoxic Cl^−^ influx was shown to be mediated by the acid‐sensitive outwardly rectifying anion channel (ASOR) in human cervical cancer HeLa cells (Wang et al., 2007) and in mouse brain cortical neurons (Sato‐Numata et al., 2014). Thus, activation of some conductive Na^+^ entry pathway must be, in parallel with ASOR activation, induced by acid stimulation upon the acidotoxic NVI event. There exist two types of known cationic channels activated by acid stimulation: one is the acid‐sensing ion channels (ASIC) mainly expressed in neuronal cells (Waldmann et al., 1997; 1999), and another is the transient receptor potential cation channel subfamily M member 7 (TRPM7) expressed in a wide variety of cell types (Jiang et al., 2005) including HeLa cells (Numata et al., 2007b; Numata and Okada, 2008b).

In this study, therefore, a possible involvement of TRPM7 in acidotoxic NVI and necrotic death in human cervical cancer HeLa cells was first investigated, not only because the normal vaginal pH is strongly acidic but also because TRPM7 is known to be aberrantly expressed in malignant tumor cells (Yee, 2017). Progesterone (P4) is known to inhibit cervical and vaginal squamous cell proliferation through mediation by the progesterone receptor (PR) (Mehta et al., 2016), and PR is known to be expressed in the endocervix epithelium (Fernandes et al., 2017). Also, progesterone was shown to inhibit cervical carcinogenesis (Yoo et al., 2013). Thus, the effects of progesterone on TRPM7 activity as well as on the acidotoxic NVI and necrosis in HeLa cells were next investigated in this study.

## Materials and Methods

### Cell culture

Human cervical cancer HeLa and human embryonic kidney HEK293T cells were grown as a monolayer in Minimum Essential Medium (MEM) and Dulbecco’s Modified Eagle's Medium (DMEM), respectively, supplemented with 10% fetal bovine serum, 40 units/mL penicillin G, and 100 *µ*g/mL streptomycin under a 95% air, 5% CO_2_ atmosphere at 37°C. For cell volume and cell size measurements, and electrophysiological experiments, cells were detached from a plastic substrate and cultured in suspension with agitation for 15–300 min.

### Recombinant expression and small interfering RNA transfection

Recombinant and expression was performed as previously described (Numata et al., 2007a). HEK293T cells were transfected with recombinant plasmids pIRES2‐EGFP vector (Clontech, Mountain View, CA), pIRES2‐EGFP‐TRPM7, pIRES2‐EGFP‐TRPM7 (D1054A), or pIRES2‐EGFP‐TRPM7 (D1054E), as previously described (Numata and Okada, 2008b). Lipofectamine 2000 (Thermo Fisher Scientific, Waltham, MA) was used for transfections according to the manufacturer’s instructions.

HeLa cells were transfected with small interfering RNA (siRNA), as previously reported (Numata et al., 2007b). For experiments, successfully transfected cells were selected by their Alexa 488 fluorescence as a transfection marker. Suppression of molecular expression of TRPM7 by the siRNA treatment in HeLa cells was confirmed by reverse transcription‐PCR and immunoblot analyses, as previously described (Numata et al., 2007b).

### Patch‐clamp experiments

Whole‐cell recordings were performed at room temperature (22–26°C). Pipettes were pulled from borosilicate glass capillaries with a micropipette puller (P‐97; Sutter Instruments, Novato, CA). The electrode had a resistance of around 4 megaohms for whole‐cell recordings when filled with pipette solution. Currents were recorded using an Axopatch 200B amplifier (Axon Instruments/Molecular Devices, Union City, CA). Current signals were filtered at 5 kHz using a four‐pole Bessel filter and digitized at 20 kHz. pClamp software (version 10.5.1.0; Axon Instruments/Molecular Devices) was used for command pulse control, data acquisition, and analysis. To minimize K^+^ and anion currents, all recordings were carried out using intracellular low Cl^−^, Cs^+^‐rich solution and extracellular Cl^−^‐free, Na^+^‐rich solution. Data were analyzed using Origin (OriginLab Corp., Northampton, MA) software. For whole‐cell recordings, the series resistance was compensated (to 70–80%) to minimize voltage errors. Ramp pulses were applied from −100 mV to +100 mV at a speed of 1 mV/msec. The intracellular (pipette) solution contained (in mmol/L): 100 Cs‐aspartate, 1 EGTA, 10 HEPES, and 0.5 CsCl (pH adjusted to 7.4 with CsOH, and osmolality adjusted to 300 mosmol/kg‐H_2_O with D‐mannitol). The bath solution contained (in mmol/L): 100 Na‐aspartate (or Cs‐aspartate), 1 CaSO_4_, 1 MgSO_4_, and 10 HEPES (pH adjusted to 7.4 with NaOH (or CsOH), and osmolality adjusted to 320 mosmol/kg‐H_2_O with D‐mannitol). To make the acidic solution (pH 4 or 6), MES was added instead of HEPES and pH was adjusted with H_2_SO_4_.

### Western blot analysis

After 72 h of application of progesterone, HeLa cells were solubilized in the radioimmunoprecipitation assay (RIPA) buffer (pH 8.0) containing 0.1% SDS, 0.5% sodium deoxycholate, 1% Nonidet P40, 150 mmol/L NaCl, 50 mmol/L Tris‐HCl, 1 mmol/L PMSF and 10 *μ*g/*μ*L leupeptin, then centrifuged at 17,400*g* for 20 min. Whole‐cell lysates were fractionated by 7.5% SDS‐PAGE and electro‐transferred onto a poly‐vinylidene fluoride (PVDF) membrane. The blots were incubated with anti‐TRPM7 antibody (1:1000 dilution, an affinity‐purified polyclonal rabbit antibody raised against a peptide corresponding to amino acids 1816–1835 of human TRPM7) or monoclonal anti‐α‐tubulin (as an internal standard, 1:2000 dilution; T6074, Sigma‐Aldrich,Saint Louis, MO), and stained using the enhanced chemiluminescence system (Thermo Fisher Scientific).

### RNA isolation and RT‐PCR

Total cellular RNA was extracted from HeLa cells using NucleoSpin®RNA Plus (Takara‐Bio, Shiga, Japan) according to the protocol supplied by the manufacturer. The concentration and purity of RNA were determined using a Nanodrop‐ND1000 (Thermo Fisher Scientific). Total RNA samples were reverse‐transcribed at 42°C for 30 min with Prime Script RTase using the Prime‐Script™ II High Fidelity RT‐PCR Kit (Takara‐Bio), according to the manufacturer’s protocols. Expression levels of TRPM7 in the cDNA from HeLa were determined by PCR. As a positive control, we amplified the partial sequence of glyceraldehyde‐3‐phosphate dehydrogenase (GAPDH). Suppression of RNA expression was confirmed by RT‐PCR analysis. PCR was done using KOD‐Plus‐Ver.2 (Toyobo, Osaka, Japan) under the following conditions: predenaturation at 94°C for 2 min, followed by 25 cycles of denaturation at 98°C for 10 sec and annealing at 55°C for 30 sec, and final extension at 68°C for 40 sec. The sequences of gene‐specific primers (synthesized by Sigma‐Aldrich) and the predicted lengths of PCR products are as follows: hGAPDH (496 bp) forward and reverse primers: 5′‐GGTGAAGGTCGGAGTCAACG‐3′ and 5′‐CAAAGTTGTCATGGATGACC‐3′, respectively; hTRPM7 (276 bp) forward and reverse primers: 5′‐CACTTGGAAACTGGAACC‐3′ and 5′‐CGGTAGATGGCCTTCTACTG‐3′, respectively.

### Cell counting assay

HeLa cells (1 × 10^5^ cells) were replated in a 6‐cm dish and incubated in 10% serum‐added MEM medium for 3 days with or without progesterone. Thereafter, an aliquot (1 *μ*L) of the Acridine Orange/Propidium Iodide (AO/PI) cell viability kit (Logos Biosystems, Inc., Anyang, Republic of Korea) was added to each 100 *μ*L of samples. After the incubation at room temperature for 10 min, the cell staining solution was loaded onto the counting slide of Countess and the loaded cell sample images were acquired from CountessII‐FL (Thermo Fisher Scientific). The cells positive for AO were taken viable and counted to calculate the proliferation rate.

### Assays for cell death

Cell death was detected by four different methods, fluometric analysis of caspase 3/7 activation, electronic sizing of cell volume, and triple staining with hoechst/acridine orange (AO) and propidium iodide (PI) assay.

Total cell caspase 3/7 activity was measured by a fluometric caspase 3/7 assay using the Caspase‐Glo® 3/7 assay systems (Promega, Madison, WI).

Mean cell volume was measured in Tyrode or Tyrode‐based acidic solution at room temperature by electronic sizing with a Coulter‐type cell size analyzer (CDA‐500; Sysmex, Hyogo, Japan). The mean volume of the cell population was calculated from the cell volume distribution measured after the machine was calibrated with latex beads of known volume. Tyrode solution (300 mosmol/kg‐H_2_O adjusted by D‐mannitol) contained (in mmol/L) 140 NaCl, 5 KCl, 1 MgCl_2_, 2 CaCl_2_, 10 D‐glucose, and 10 HEPES (pH 7.4 adjusted by NaOH). To make the Tyrode‐based acidic solution (pH 4 or 6), MES was added instead of HEPES and pH was adjusted with H_2_SO_4_. The relative cell volume is defined by the following equation: relative cell volume = *V*_A_/*V*_0_, where *V*_0_ and *V*_A_ are the mean cell volume values at an initial and a given time during the experiments. The mean cell size was measured at room temperature by video image recording. The EGFP‐positive cells were visualized through a charge‐coupled device camera (XC‐ST70, Sony, Tokyo, Japan) and images were recorded with the mAgicTV software (I‐O DATA, Ishikawa, Japan). The cross‐sectional area (CSA) of the cell of interest was measured as an indicator of cell size by the ImageJ software. The relative CSA is defined by the following equation: relative CSA = *A*/*A*_0_, where *A*_0_ and *A* are the CSA values at an initial and a given time, respectively, during the experiments.

For morphological analysis of nuclei, and triple staining with hoechst/acridine orange (AO) and propidium iodide (PI) assay, the Hoechst 33342 (2 *μ*g/mL), and propidium iodide (PI: 5 *μ*g/mL)/AO/PI cell viability kit (Logos Biosystems) were added to each sample. After the incubation at room temperature for 5 min, the cell sample images were acquired in EGFP‐positive cells from In Cell Analyzer 1000 (GE Healthcare, CA)/ CountessII‐FL (Thermo Fisher Scientific). The PI‐positive cells were taken as necrotic cells and counted. In those assays, cells were preincubated in acidic solution for 1 h and washed with PBS. The composition of acidic solutions was the same as that of acidic solutions used in cell volume measurements.

### Statistical evaluation

Data are presented as means ± SEM of *n* observations. Statistical differences of the data were evaluated by the paired or unpaired Student’s *t* test and were considered significant at *P* < 0.05.

## Results

### TRPM7 expression is involved in acid‐induced necrotic cell death in human cervical cancer HeLa cells

**Figure 1:**
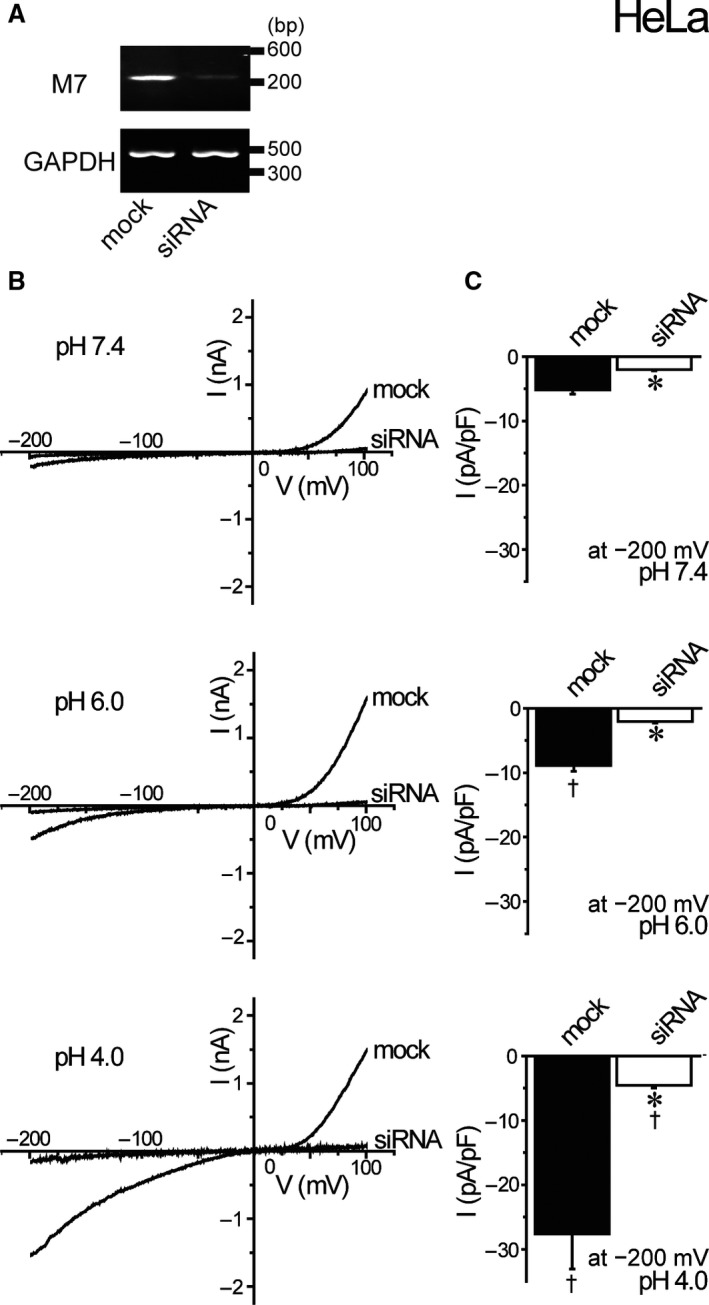
Abrogating effects of siRNA knockdown of TRPM7 gene on acid‐induced whole‐cell currents in HeLa cell. (A) Detection of TRPM7 and GAPDH mRNAs by RT‐PCR. Transfection with TRPM7‐siRNA for 72 h suppressed TRPM7 mRNA expression (siRNA: compared to mock) without affecting GAPDH mRNA expression. Data are representative of duplicate experiments. (B) Representative current–voltage (*I*–*V*) relationships of cationic currents at pH 7.4, pH 6.0, and pH 4.0 under ramp clamp from −200 to +100 mV in mock‐ or siRNA‐treated cell. (C) Mean current densities recorded at −200 mV of mock‐ or siRNA‐transfected cells in pH 7.4, pH 6.0, and pH 4.0 solutions (*n* = 10). Each data point represents the mean ± SEM. *Significantly different (*P* < 0.05) from the corresponding control (mock‐transfected) cells. ^†^Significantly different (*P* < 0.05) from the values at pH 7.4.

HeLa cells are known to express TRPM7 activity which exhibits outwardly and inwardly rectifying cation currents (Numata et al., 2007b) and is enhanced by acidic solution (Numata and Okada, 2008a). Actually, the treatment with siRNA for TRPM7 mRNA (TRPM7‐siRNA) markedly suppressed TRPM7 expression (Fig. 1A) and largely eliminated cationic currents observed at pH 7.4 and also acid‐enhanced cationic currents (Fig. 1B and C), indicating that cationic currents enhanced by exposure to acidic solution in HeLa cells are mediated via TRPM7 under the present experimental conditions.

**Figure 2:**
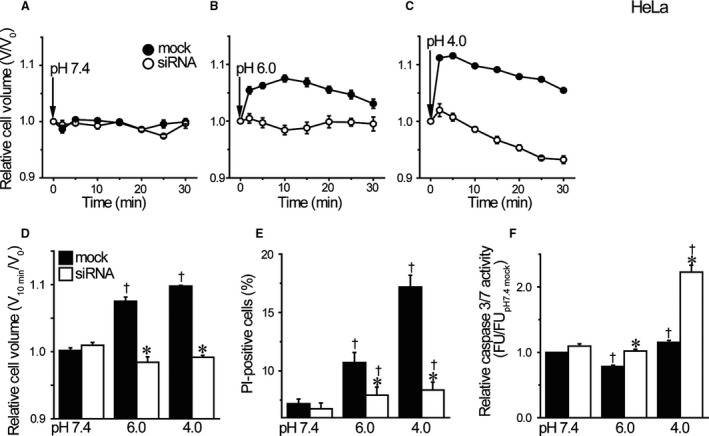
Effects of TRPM7 knockdown on acid‐induced cell swelling and death in HeLa cells. (A‐C) The time course of changes in the relative mean cell volume. At time zero, pH of the bathing solution was changed. Acidic solution (pH 6.0 or 4.0) induced the necrotic volume increase (NVI) in mock‐transfected, but not TRPM7‐siRNA‐transfected, HeLa cells (*n* = 10). (D) Summarized data showing the relative cell volume at 10 min after application of acidic solution (pH 6.0 or 4.0). Each column represents the mean ± SEM. (E) Summarized data showing the fraction of PI‐positive cells after 1‐h exposure to pH 7.4 solution or acidic solution (pH 6.0 or 4.0) in mock‐transfected control cells (filled columns) or TRPM7‐siRNA‐transfected cells (open columns). Each column represents the mean ± SEM (*n* = 19). (F) The relative caspase 3/7 activity measured by a fluorometric assay after 1‐h exposure to pH 7.4 or acidic solution. Each column represents the mean ± SEM in five independent experiments. *Significantly different (*P* < 0.05) from the corresponding control (mock‐transfected) cells. ^†^Significantly different (*P* < 0.05) from the values at pH 7.4.

As shown in Figure 2A–C, HeLa cells responded to exposure to acidic solution at pH 6.0 and pH 4.0 with cell swelling reaching the mean peak values of 108% and 110%, respectively, within 10 min (Fig. 2D), as previously observed at pH 4.5 (Wang et al., 2007). Acid‐induced cell swelling was abolished in HeLa cells treated with TRPM7‐siRNA (Fig. 2A–D). Interestingly, the cell volume observed at 30 min after exposure to pH 4.0 acidic solution in siRNA‐treated cells significantly showed cell shrinkage (Fig. 2C: open symbols). Triple staining experiments with Hoechst 33342, acridine orange and propidium iodide (PI) showed that the number of PI‐positive (dead) cells markedly increased after 1‐h exposure to acidic solutions at pH 6.0 and 4.0 in mock‐transfected control HeLa cells (Fig. 2E: filled columns), whereas acid‐induced cell death became much less marked in TRPM7‐deficient HeLa cells (Fig. 2E: open columns). As shown in Figure 2F, acid treatment induced activation of apoptotic caspase 3/7 in TRPM7‐deficient cells (open columns), being in contrast to little caspase 3/7 activation in control cells (filled columns). These results indicate that acid exposure induced cell swelling followed by PI‐positive cell death in TRPM7‐expressing cells but rather cell shrinkage followed by PI‐negative cell death characterized by caspase 3/7 activation in TRPM7‐deficient cells. Thus, it appears that the expression of TRPM7 is required for the acid‐induced necrotic volume increase (NVI) and necrotic cell death in HeLa cells, and that when the expression of TRPM7 is suppressed by siRNA treatment, acid exposure rather induced cell shrinkage, that is the apoptotic volume decrease (AVD), and PI‐negative cell death associated with caspase 3/7 activation, that is apoptosis.

### TRPM7 conductance is involved in acid‐induced necrotic cell death in TRPM7‐overexpressing human epithelial HEK293T cells

**Figure 3:**
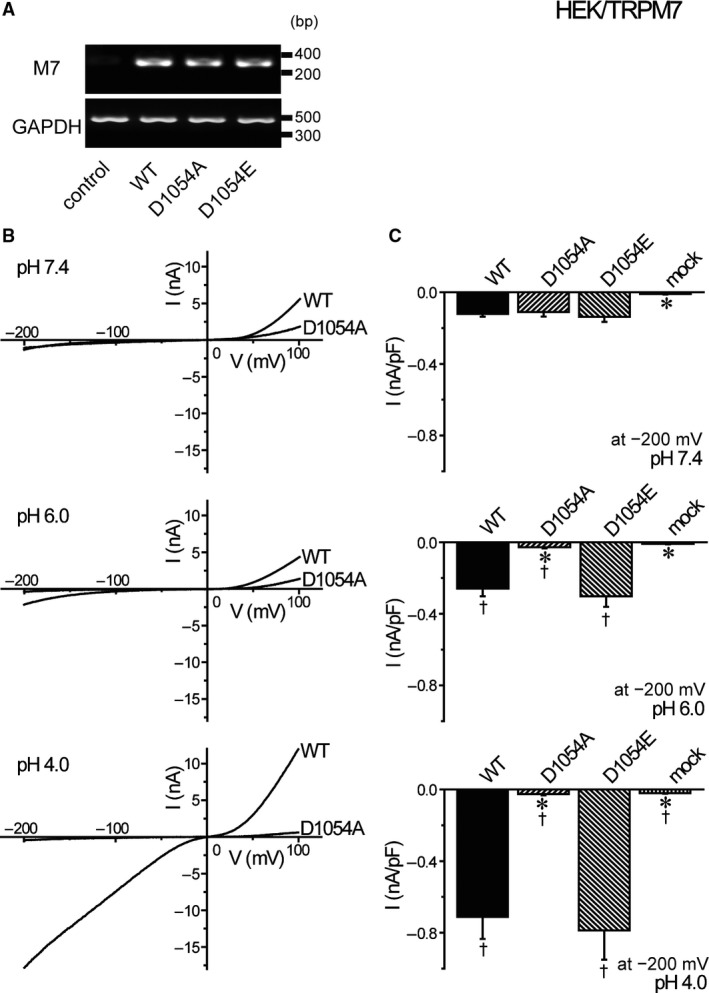
Effects of overexpression of WT and mutant TRPM7 on acid‐induced whole‐cell currents in HEK293T cells. (A) Detection of TRPM7 and GAPDH mRNAs by RT‐PCR. Transfection with WT, D1054A, and D1054E of TRPM7 enhanced TRPM7 mRNA expression (compared to mock) without affecting GAPDH mRNA expression. Data are representative of duplicate experiments. (B) Representative *I*–*V* relationships of cationic currents at pH 7.4, pH 6.0, and pH 4.0 under ramp clamp from −200 to +100 mV in WT‐ or D1054A‐transfected cells. (C) Mean current densities at −200 mV of mock‐, WT‐, D1054A‐, or D1054E‐transfected cells in pH 7.4, pH 6.0, and pH 4.0 solutions (*n* = 6–14). Each data point represents the mean ± SEM. *Significantly different (*P* < 0.05) from the corresponding WT‐transfected data. ^†^Significantly different (*P* < 0.05) from the values at pH 7.4.

A pore charge‐neutralizing D1054A mutant, but not a charge‐preserving mutant D1054E, of TRPM7 is known to inhibit cation currents under strong acidic conditions (Numata and Okada, 2008a). To confirm the involvement of TRPM7‐mediated cation conductance in acid‐induced necrotic cell death, therefore, effects of acid exposure on the channel activity and necrotic cell death were examined in HEK293T cells overexpressing the wild‐type (WT) and D1054A and D1054E mutants of TRPM7 after validating their successful overexpression (Fig. 3A). As shown in Figure 3B and C, exposure to acidic solution was found to enhance whole‐cell currents exhibiting outwardly and inwardly rectifying currents typical to TRPM7 channel activity in HEK293T cells transfected with wild‐type TRPM7 (WT) but not in the mock‐transfected cells (mock). When the D1054A mutant was overexpressed, acid‐induced current activation was mostly abolished (D1054A), whereas the D1054E mutant failed to affect acid‐induced current increments (D1054E).

**Figure 4:**
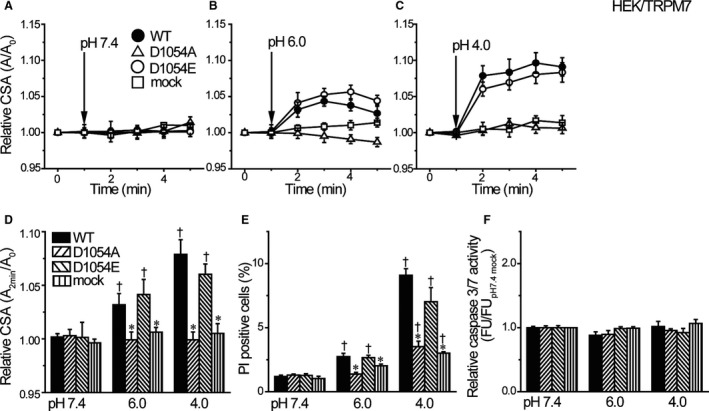
Effects of overexpression of TRPM7 and its mutants on acid‐induced cell swelling and death. (A–C) The time course of changes in the mean relative cross‐sectional area (CSA). At 1 min, pH of the bathing solution was changed. Acidic solution (pH 6.0 or 4.0) induced the necrotic volume increase (NVI) in WT‐ and D1054E‐transfected, but not in mock‐ and D1054A‐transfected, HEK293T cells (*n* = 10). (D) Summarized data showing the relative CSA at 2 min after application of acidic solution (pH 6.0 or 4.0). (E) Summarized data showing the fraction of PI‐positive cells after 1‐h exposure to pH 7.4 solution or acidic solution (pH 6.0 or 4.0) in WT‐, D1054A‐, D1054E‐ or mock‐transfected cells. (F) The relative caspase 3/7 activity measured by a fluorometric assay after 1‐h exposure to pH 7.4 or acidic solution. Each column represents the mean ± SEM in five independent experiments. *Significantly different (*P* < 0.05) from the corresponding WT‐transfected data. ^†^Significantly different (*P* < 0.05) from the values at pH 7.4.

In HEK293T cells overexpressing WT and pore mutants of TRPM7, the cell size was maintained constant at pH 7.4 (Fig. 4A). In contrast, as seen in Figure 4B and C, the WT‐expressing HEK293T cells responded to acidic solutions at pH 6.0 and pH 4.0 with cell swelling (filled circles). However, acid‐induced cell swelling was not observed in cells expressing the D1054A mutant (open triangles) or mock‐transfected control cells (open squares). On the other hand, in the cells expressing the D1054E mutant, acid exposure induced cell swelling (open circles), as observed in WT‐expressing cells (filled circles). As summarized in Figure 4D, acid‐induced cell swelling was significantly observed in WT‐ and D1054E‐expressing cells but not in D1054A‐expressing and mock‐transfected cells. Acid treatment increased the number of PI‐positive cells in WT‐ and D1054E‐expressing cells, whereas such acid‐induced cell death was much less prominent in D1054A‐ and mock‐transfected cells (Fig. 4E). In contrast, exposure to acidic solution never induced caspase 3/7 activation in WT‐, D1054A‐, D1054E‐ and mock‐transfected cells (Fig. 4F). Thus, it is evident that activation of TRPM7 conductance is responsible for acid‐induced NVI and necrotic cell death by causing Na^+^ influx and inducing depolarization facilitating Cl^−^ influx via the anionic ASOR conductance in the human epithelial cells.

### Progesterone inhibits molecular and functional TRPM7 expression and suppresses cell proliferation in human cervical cancer HeLa cells

**Figure 5:**
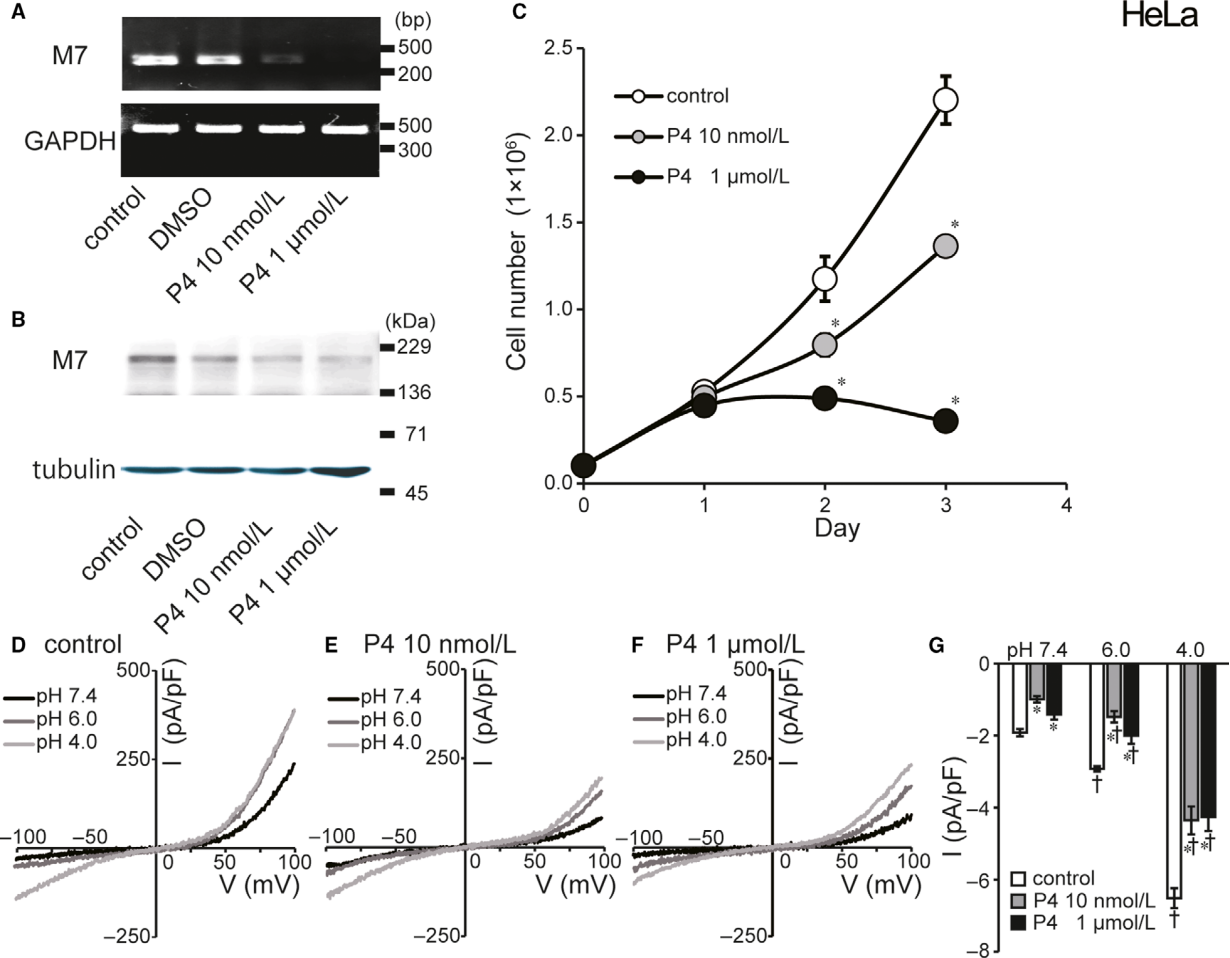
Effects of progesterone (P4) treatment on molecular and functional expression of TRPM7 and cell proliferation in HeLa cell. (A) Detection of TRPM7 and GAPDH mRNAs by RT‐PCR. Treatment with progesterone (10 nmol/L or 1 *µ*mol/L) for 72 h suppressed TRPM7 mRNA expression (compared to DMSO‐treated or untreated control) without affecting GAPDH mRNA expression. Data are representative of duplicate experiments. (B) Immunoblots of the protein extracts of HeLa cells for TRPM7 protein. Progesterone treatment reduced expression of TRPM7 protein. Data are representative of triplicate experiments. (C) Effects of progesterone on cell proliferation evaluated at 1, 2, and 3 days after culture. Gray and filled circles represent the number of HeLa cells treated with 10 nmol/L or 1 *µ*mol/L progesterone, respectively. Open circles represent the number of control cells. (D, E, F) Representative *I–V* relationships of cationic currents under ramp clamp from −100 to +100 mV in untreated control and progesterone‐treated cells. (G) Summarized data showing the whole‐cell current densities recorded at −100 mV in untreated control cells (white columns), 10 nmol/L (gray columns) and 1 *µ*mol/L progesterone‐treated cells (black columns). Acid treatment (pH 6.0 or 4.0) significantly reduced whole‐cell currents compared to untreated control cells (*n* = 7–15). Each column represents the mean ± SEM (vertical bar). *Significantly different (*P* < 0.05) from the control values. †Significantly different (*P* < 0.05) from the values at pH 7.4.

As shown in Figure 5 (A and B), administration of progesterone (P4: 10 nmol/L or 1 *µ*mol/L for 72 h) was found to drastically reduce the endogenous expression of TRPM7 mRNA (A) and protein (B) in HeLa cells. Since TRPM7 is known to play an essential role in cell proliferation (Hanano et al., 2004; Jiang et al., 2007), we then explored the effect of progesterone on the proliferation of HeLa cells. As shown in Figure 5C, the proliferation of HeLa cells was markedly suppressed by 10 nmol/L progesterone and almost completely ceased by 1 *µ*mol/L progesterone. Whole‐cell recordings in HeLa cells showed that cationic currents activated by acidic solution (Fig. 5D and G) were found to be significantly reduced after progesterone administration (10 nmol/L and 1 *µ*mol/L: Fig. 5E–G).

### Progesterone inhibits TRPM7‐mediated acidotoxic necrosis in human cervical cancer HeLa cells

**Figure 6:**
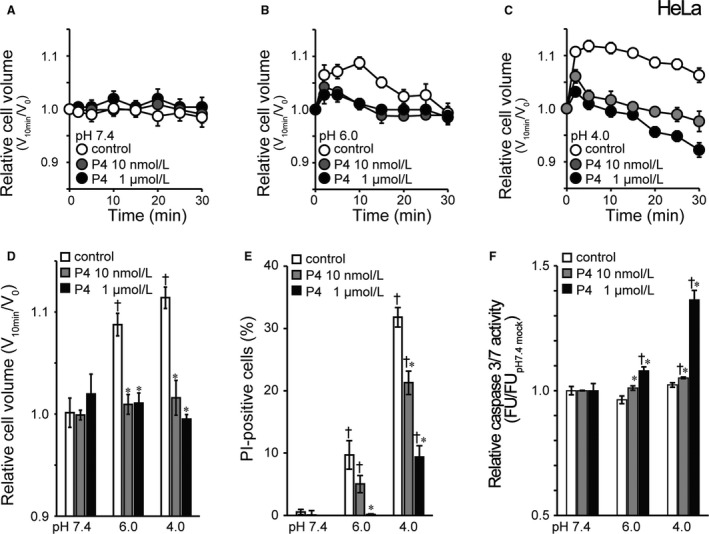
Switching effects of treatment with progesterone (P4) on acid‐induced cell death from necrosis to apoptosis in HeLa cells. (A–C) The time course of changes in the relative mean cell volume. At time zero, pH of the bathing solution was changed. Acidic solution (pH 6.0 or 4.0) induced the necrotic volume increase (NVI) in untreated control, but not progesterone‐treated, cells (*n* = 5). (D) Summarized data showing the relative cell volume at 10 min after application of acidic solution (pH 6.0 or 4.0). (E) Summarized data showing the fraction of PI‐positive cells after 1‐h exposure to pH 7.4 solution or acidic solution (pH 6.0 or 4.0) in untreated control cells (white columns) or cells treated with progesterone at 10 nmol/L (gray columns) and 1 *µ*mol/L (black columns). Each column represents the mean data in six different experiments. (F) The relative caspase 3/7 activity measured by a fluorometric assay after 1‐h exposure to pH 7.4 or acidic solution. Each column represents the mean ± SEM in eight independent experiments. *Significantly different (*P* < 0.05) from the corresponding control cells. ^†^Significantly different (*P* < 0.05) from the values at pH 7.4.

At pH 7.4 the cell volume was virtually constant, and 72‐h treatment with 10 nmol/L and 1 *µ*mol/L progesterone did not affect the HeLa cell volume (Fig. 6A). In HeLa cells treated with 10 nmol/L and 1 *µ*mol/L progesterone for 72 h, acid‐induced cell swelling was abolished at pH 6.0 (Fig. 6B), as summarized in Figure 6D, and even turned to shrinkage at 30 min after application of acidic solution at pH 4.0 (Fig. 6C). These progesterone effects are similar to the effects of TRPM7‐siRNA (Fig. 2D). PI‐positive HeLa cells observed after 1‐h exposure to acidic solution at pH 6.0 were abolished by treatment with 1 *µ*mol/L progesterone and markedly reduced at pH 6.0 by treatment with 10 nmol/L progesterone and at pH 4.0 by treatment with 10 nmol/L and 1 *µ*mol/L progesterone, as shown in Figure 6E (gray and black columns). As summarized in Figure 6F (gray and black columns), caspase 3/7‐positive cells became observed by treatment with 10 nmol/L progesterone at pH 4.0 and by treatment with 1 *µ*mol/L progesterone at pH 6.0 and 4.0. Thus, it appears that apoptosis was the predominant cell death induced by acid exposure in progesterone‐treated cells, but necrotic cell death predominated under untreated control conditions in HeLa cells. On balance, the progesterone treatment turns the acid‐induced volume change from NVI to AVD and cell death from apoptosis to necrosis by suppressing TRPM7 expression in the human cervical cancer cells.

## Discussion

TRPM7 has been implicated in a wide variety of physiological cellular processes, including cell proliferation (Hanano et al., 2004; Jiang et al., 2007), divalent cation homeostasis (Monteilh‐Zoller et al., 2003; Schmitz et al., 2003; He et al., 2005; Inoue et al., 2010), cytoskeletal regulation (Nadler et al., 2001; Clark et al., 2006), cell adhesion (Su et al., 2006), cell migration (Wei et al., 2009; Kuras et al., 2012), axonal outgrowth (Turlova et al., 2016), neurotransmitter release (Krapivinsky et al., 2006; Brauchi et al., 2008), embryonic development (Jin et al., 2008), mechano‐sensing (Numata et al., 2007a,2007b), and intestinal pace‐making (Kim et al., 2005). Involvements of TRPM7 were also shown in hypoxic/ischemic brain injury and neuronal cell death (Aarts et al., 2003; Aarts and Tymianski, 2005; Jiang et al., 2008; Sun et al., 2009; Leng et al., 2014; Chen et al., 2015) and Zn‐induced neurotoxicity (Inoue et al., 2010). Furthermore, TRPM7 is known to be aberrantly expressed in human cancers and playing roles in tumor growth and metastasis (Yee, 2017). In this study, TRPM7 channel activity was, for the first time, demonstrated to play an essential role in the acidotoxic induction of NVI and necrotic cell death in human cervical cancer HeLa cells.

Progesterone (P4), which belongs to a group of steroid hormones, is essential for the regulation of normal female reproductive functions with mainly regulating the monthly menstrual cycle and maintaining the early stages of pregnancy in the uterus and ovary. The physiological effects of progesterone are mediated via the interaction of the hormone with two progesterone receptor (PR) isoforms, PR‐A and PR‐B. PR is known to express in the epithelium of the endocervix (Fernandes et al., 2017), suppress cervical cell proliferation (Mehta et al., 2016) and inhibit cervical carcinogenesis (Yoo et al., 2013). However, the effects of progesterone on cervical cancer cells have not as yet been well elucidated especially under the acidic conditions mimicking the intravaginal situation. In human cervical cancer HeLa cells, here, for the first time, we demonstrated that progesterone reduces the endogenous expression of TRPM7 molecule (Fig. 5A and B) and its channel activity (Fig. 5D–G) as well as the cell proliferation (Fig. 5C). Also, progesterone treatment was found to inhibit acid‐induced cell swelling (Figs. 6B–D) and acid‐induced necrotic cell death (Fig. 6E) with activating apoptotic caspase 3/7 (Fig. 6F). These results strongly suggest that progesterone inhibits the proliferation of cervical cancer cells and switches acidotoxic cell death from necrosis to apoptosis by suppressing TRPM7 expression and activity.

Apoptosis is the process by which cells are programmed to die in an orderly and timely manner and also the prerequisite process to promptly remove dying and damaged cells (Wyllie et al., 1980; Raff et al., 1994). Cancer cells, in contrast, escape from the apoptotic cell death by acquiring a dysfunctional apoptotic program (Hacker and Vaux, 1995; Thompson, 1995; Renehan et al., 2001). Thus, a variety of anticancer agents were designed to induce activation of apoptotic program (Kaufmann, 1989; Barry et al., 1990; Eastman 1990). Cancer cells are in general induced to die with non‐apoptotic mechanism such as necrosis (Okada and Mak, 2004). In necrosis, cytosolic and nuclear constituents leak out to the intercellular space through the broken or damaged plasma membrane, thereby provoking inflammatory response in the surrounding tissues. In contrast, apoptosis is coupled to phagocytosis by nearby phagocytes to clear dying cells and apoptotic bodies without scattering harmful intracellular constituents, namely damage‐associated molecular patterns (DAMPs), derived from dying cells. Thus, an anticancer strategy is to be targeting the switching method of necrosis to apoptosis.

Cervical cancer is a serious problem with nearly 500,000 women developing the disease each year (Waggoner, 2003) and with resulting in the fourth leading cause of cancer death in females worldwide (Jemal et al., 2011). Although the surgical operation and chemo‐radiotherapy are most effective to cure the patients with the early stage (say Stage 0 to IB) and the middle stage (say Stage IIA to IVA) of cervical cancer, respectively, the latter therapy often produces hematologic and gastrointestinal side effects (Eifel, 2006). Progesterone and its derivative progestines have been used successfully as therapeutics to treat endometriosis and endometrial cancer (for Review: (Kim et al., 2013) but not cervical cancer. The present finding of progesterone‐induced necrosis‐to‐apoptosis switching under acidic conditions might provide an important clue for a possible application of the progesterone therapy as an additional therapeutic intervention for the middle to late stages of cervical cancer, when taking the acidic situations not only of the cervical and vaginal fluids but also of the solid tumor microenvironment into consideration.

## Conflict of Interest

No potential conflicts of interest were disclosed.
